# Ferroptosis in Adipose Tissue: A Double‐Edged Sword Between Obesity Therapy and Stem Cell Risk

**DOI:** 10.1002/mco2.70512

**Published:** 2025-11-20

**Authors:** Mohammed Zayed, Byung‐Hoon Jeong

**Affiliations:** ^1^ Korea Zoonosis Research Institute Jeonbuk National University Iksan Republic of Korea; ^2^ Department of Bioactive Material Sciences Jeonbuk National University Jeonju Republic of Korea; ^3^ Department of Surgery College of Veterinary Medicine South Valley University Qena Egypt

**Keywords:** adipose tissue, ferroptosis, obesity treatment, stem cells

1

In a recent study published in *Cell Metabolism*, Wang et al. highlighted the therapeutic potential of activating ferroptosis to reduce lipid accumulation [1]. Their work demonstrated that activating ferroptosis with non‐lethal agonists can significantly reduce lipid accumulation in adipocytes and obese mice. However, this potential therapy represents a double‐edged sword, as inducing ferroptosis may damage adipose‐derived stem cells (ADSCs), impairing their essential regenerative functions like proliferation and differentiation. This underscores the critical balance between therapeutic efficacy and stem cell safety.

Obesity is characterized by the excessive accumulation of adipose tissue. This tissue is a dynamic endocrine organ crucial in regulating metabolism and maintaining homeostasis. Its function in health and metabolic diseases is affected by cellular composition, the array of molecules it secretes, and anatomical location. Given that the dysregulation of cell death pathways is a hallmark of dysfunctional adipose tissue in obesity, a novel form of regulated cell death, ferroptosis, has recently gained substantial interest. Ferroptosis is a regulated cell death driven by iron‐dependent lipid peroxidation [2]. Significant advancements have been made in developing pharmacological agonists and antagonists for treating conditions related to ferroptosis. Wang et al. highlighted a new therapeutic approach to reduce obesity through controlled induction of ferroptosis with the agonist RAS‐selective lethal 3 (RSL3). Topical application of RSL3 increased lipid peroxidation and ferroptotic signaling in the white adipose tissue of mice fed a high‐fat diet, thereby reducing lipid accumulation (Figure 1A,B). This treatment alleviates obesity and related metabolic disorders, suggesting that inducing ferroptosis could offer a novel approach to managing obesity. However, this therapeutic potential raises a crucial safety concern: how does induced ferroptosis affect the viability and function of other cellular populations within adipose tissue, especially ADSCs, a key cell population within adipose tissue?

**FIGURE 1:**
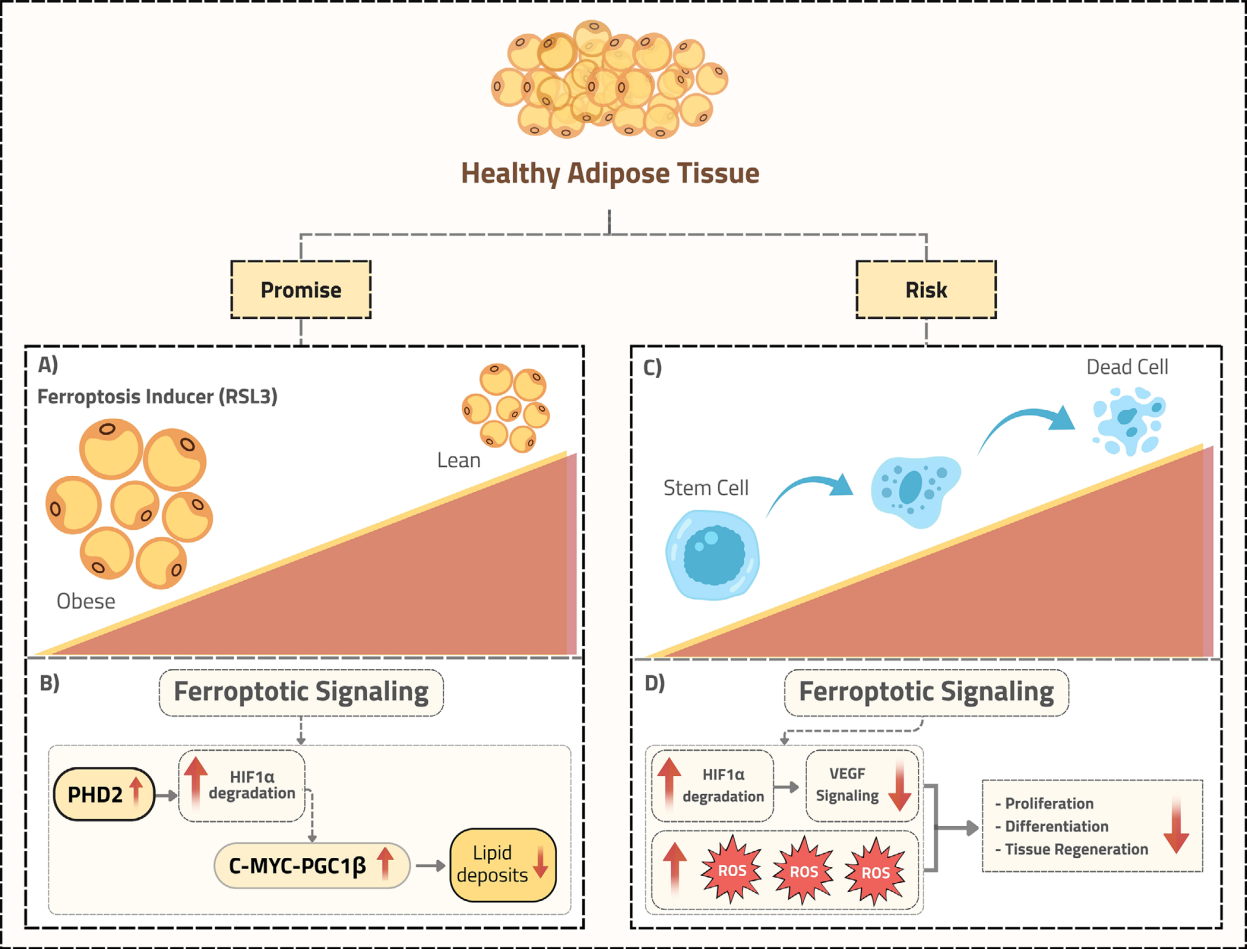
Ferroptosis in adipose tissue offers therapeutic potential but also poses a stem cell risk. This schematic illustrates the double‐edged sword of inducing ferroptosis in adipose tissue. (A, B) The left panel (Promise) explains the therapeutic approach for obesity: Pharmacologically inducing ferroptosis (using Ras‐selective lethal 3, RSL3) in adipocytes increases prolyl hydroxylase domain 2 (PHD2) levels, leading to degradation of hypoxia‐inducible factor 1α (HIF1α). This process boosts a thermogenic program regulated by the c‐MYC‐peroxisome proliferator‐activated receptor gamma coactivator‐1 beta (PGC1β) pathway, decreasing adipose tissue [1]. (C, D) The right (Risk) panel highlights the primary safety concern regarding adipose‐derived stem cells within the same tissue. Ferroptosis inducers increase reactive oxygen species (ROS) and cause HIF1α degradation, disrupting proliferation, differentiation, and overall regenerative capacity, significantly threatening tissue homeostasis.

ADSCs have been introduced as an alternative to bone marrow‐derived stem cells (BMSCs) for cell‐based therapy due to their high proliferation and differentiation capacity. They can be obtained in large quantities using a minimally invasive procedure, making them an outstanding source for tissue regeneration [3]. Given their therapeutic importance, a key question arises: Are ADSCs more susceptible to ferroptosis than other stem cells, such as BMSCs, and how might this affect their regenerative potential? ADSCs reside in the lipid‐rich environment of adipose tissue, facilitating their abundance and ease of isolation. However, this unique microenvironment may increase their susceptibility to ferroptosis. If ADSCs are severely affected, their regenerative potential could be compromised, particularly in contexts where ferroptosis is intentionally induced to treat obesity. It has been reported that the susceptibility of cells to ferroptosis is closely tied to their lipid content and composition, as lipid peroxidation is a crucial feature of this process [2]. Unlike BMSCs, which reside in the bone marrow, a niche rich in hematopoietic and osteogenic cells but low in lipids, ADSCs reside in adipose tissue, surrounded by lipid‐storing adipocytes [3]. Furthermore, studies have found that ADSCs have higher intracellular lipid levels, reflecting their origin and adipogenic potential [4]. These lipids, particularly polyunsaturated fatty acids, are highly prone to peroxidation due to the increased presence of peroxidation substrates [2]. Thus, the PUFA‐rich environment of adipose tissue likely increases the inherent risk of ferroptosis in ADSCs. Beyond their inherently lipid‐rich profile, ADSCs show multiple susceptibilities to ferroptosis. This predisposition arises from weaker antioxidant defenses, dysregulated iron homeostasis that enhances the labile iron pool, and PPARγ‐driven transcriptional programming that promotes the accumulation of peroxidation‐sensitive PUFAs. These factors create a perfect storm, rendering ADSCs vulnerable to ferroptotic cell death.

Furthermore, Wang et al. observed a reduced ferroptotic signature in obesity, suggesting that cells, including ADSCs, might adapt to resist ferroptosis [1]. In obesity, increased oxidative stress and altered lipid profiles may increase susceptibility to ferroptosis. To counter this, cells might employ protective mechanisms, such as upregulating ferroptosis suppressors like glutathione peroxidase 4, a critical antioxidant enzyme that neutralizes lipid hydroperoxides, or adjusting lipid metabolism to reduce peroxidation risks. These adaptations likely serve as survival strategies in a metabolically challenging environment. However, therapeutically inducing ferroptosis could disrupt these protective mechanisms in ADSCs. This disruption might lead to ADSC dysfunction or death, reducing their therapeutic potential (Figure 1C,D). Further complicating concerns, Wang et al. found that ferroptosis induction triggers the degradation of hypoxia‐inducible factor 1α (HIF1α), a protein vital for stem cell maintenance and differentiation (Figure 1B).

HIF1α degradation disrupts glycolytic metabolism, which is essential for ADSC proliferation and survival [5]. This leads to downregulating important markers such as vascular endothelial growth factor. This disruption impairs the controlled differentiation process, leading to ADSC multipotency and regenerative capacity loss. Collectively, these effects suggests that ferroptosis induction not only directly affects ADSCs through lipid peroxidation but also indirectly impairs their regenerative capacity via HIF1α loss (Figure 1D).

The regenerative capacity of ADSCs, including proliferation, differentiation, and tissue repair, is important for their therapeutic potential in regenerative medicine [3]. While their regenerative potential is compromised in obesity, these cells can still be used in regenerative therapies by enhancing their function. Thus, obesity impairs ADSCs but doesn't completely rule out their use. If ADSCs are more susceptible to ferroptosis, their potential in regenerative medicine may be compromised, particularly in environments with induced ferroptosis or high oxidative stress. For example, in obesity treatment, ferroptosis agonists might reduce adipose tissue mass but simultaneously deplete or impair ADSCs, reducing their viability and differentiation potential. Additional compromise due to ferroptosis could limit their utility, posing challenges for patients requiring tissue repair. In contrast, BMSCs may be less susceptible to ferroptosis due to their lower lipid content and potentially stronger antioxidant defenses, allowing them to maintain regenerative potential under similar conditions. This difference could shift preferences toward BMSCs in certain stem cell therapies. However, the advantages of ADSCs, including large quantities and less invasive harvesting methods, emphasize the need to address their ferroptosis‐related susceptibilities to preserve their clinical utility.

While the study by Wang et al. opens new avenues for obesity treatment through ferroptosis, its implications for ADSCs require further exploration. Ferroptosis could reduce adipose tissue, but it may also harm ADSCs. The therapeutic induction of ferroptosis in adipose tissue must be balanced against risks to the stem cell population. Quantifying the ferroptosis sensitivity of ADSCs versus other stem cells under controlled conditions is crucial for assessing differential risks and ensuring treatment safety. Furthermore, evaluating how ferroptosis inducers affect the viability, proliferation, and differentiation of ADSCs in regenerative medicine models will clarify their practical impact. Such studies will explain whether ferroptosis‐based therapies can coexist with ADSC applications or if alternative approaches are necessary to preserve their therapeutic potential.

In conclusion, the study by Wang et al. highlights ferroptosis as a promising target for obesity management while revealing a potential challenge for ADSCs. Their increased susceptibility, driven by a lipid‐rich environment, reduced antioxidant capacity, and dependence on HIF1α, suggests that ferroptosis‐based therapies could impair their regenerative capacity. Given the important role of ADSCs in regenerative medicine, targeted research is essential to optimize ferroptosis induction strategies, ensuring they enhance metabolic health without compromising the therapeutic potential of ADSCs.

## Author Contributions

M.Z. drafted the manuscript and prepared the figure. B‐H.J. drafted and reviewed the manuscript. All authors have read and approved the final manuscript.

## Ethics Statement

The authors have nothing to report.

## Conflicts of Interest

The authors declare no conflicts of interest.

## Data Availability

The authors have nothing to report.
